# Supplementary material for: Cognitive Impairment in Nonagenarians: Potential Metabolic Mechanisms Revealed by the Synergy of In Silico Gene Expression Modeling and Pathway Enrichment Analysis
Source: Int J Mol Sci. 2024 Mar 15;25(6):3344. doi: 10.3390/ijms25063344 (PMC10970146; doi:10.3390/ijms25063344)
Supplement: Supplementary file 1 [file ijms-25-03344-s001.zip › ijms-2871012-supplementary.pdf]

Supplement

Table S1. TWAS results.

Gene	Z-score	p-value	Significant
Amigdala			
PTPN22	-3.910881	0.000092	yes
ADAM33	3.667582	0.000245	no
PEX13	3.535061	0.000408	no
PHTF1	3.514200	0.000441	no
ACER2	-3.487440	0.000488	no
Anterior cingulate cortex			
LRRC25	-4.236971	0.000040	yes
PRB2	3.698503	0.000217	no
DLX6	3.582232	0.000341	no
CSF2RB	3.397982	0.000679	no
FAM120AOS	-3.394392	0.000688	no
Caudate basal ganglia			
PHTF1	3.837777	0.000124	no
CPNE7	-3.802630	0.000143	no
PSMG3	-3.690272	0.000224	no
PEX13	3.638209	0.000275	no
DLX6	3.553400	0.000380	no
Cerebellar Hemisphere			
HSPBP1	3.695897	0.000219	no
PSMG3	-3.636988	0.000276	no
SH2D7	-3.622768	0.000291	no
PHTF1	3.514200	0.000441	no
SH3BP5L	-3.509282	0.000449	no
Cerebellum			
LRRC25	-4.215465	0.000032	yes
PHTF1	3.514200	0.000441	no
RP11-560J1.2	-3.473626	0.000513	no
CPSF2	-3.370074	0.000751	no

SHF	3.348304	0.000813	no
Cortex			
LRRC25	-4.117557	0.000038	yes
SHF	-3.668321	0.000244	no
DUOX1	-3.647572	0.000265	no
PEX13	3.638209	0.000275	no
DLX6	3.582232	0.000341	no
Frontal Cortex			
TMEM14A	3.622834	0.000291	no
DLX6	3.582232	0.000341	no
PEX13	3.535061	0.000408	no
RP11-560J1.2	-3.466095	0.000528	no
LINC00390	-3.386918	0.000707	no
Hippocampus			
AP1S3	-3.551922	0.000382	no
PHTF1	3.514200	0.000441	no
PRB2	3.489408	0.000484	no
DUOX1	-3.342924	0.000829	no
ADAM33	3.250358	0.001153	no
Hypothalamus			
LRRC25	-4.001752	0.000055	yes
PRB2	3.792066	0.000149	no
DLX6	3.582232	0.000341	no
PEX13	3.535061	0.000408	no
ELANE	-3.523711	0.000426	no
Nucleus accumbens basal ganglia			
DUSP26	3.657454	0.000255	no
PEX13	3.638209	0.000275	no
AC016995.3	-3.615122	0.000300	no
DLX6	3.582232	0.000341	no
TMEM14A	3.516764	0.000437	no
Putamen basal ganglia			

RASA1	-3.847408	0.000119	no
PSMG3	-3.732257	0.000190	no
DLX6	3.582232	0.000341	no
NUPR2	3.538227	0.000403	no
PEX13	3.535061	0.000408	no
Spinal cord cervical c-1			
LRRC25	-4.116764	0.000061	yes
PHTF1	3.837777	0.000124	no
PSMG3	-3.700981	0.000215	no
ALKBH1	3.671495	0.000241	no
SH3BP5L	-3.504508	0.000457	no
Substantia nigra			
LRRC25	-4.102217	0.000092	yes
SH2D7	-3.705672	0.000211	no
PHTF1	3.514200	0.000441	no
C1orf123	3.417137	0.000633	no
RP11-560J1.2	-3.392242	0.000693	no

Note: Table presents 5 most significant genes in each brain part. Z-score>0 reflects upregulation of the gene and Z-score<0 reflects downregulation. The gene is significantly associated with cognitive impairment when p-value<10^{-4}.

Table S2. TWAS results for genes in the HALLMARK_PEROXISOME functional pathway.

Gene	Z-score
PEX13	3.63820886611938
CLN6	2.67555570602417
PRDX1	2.1304612159729
CAT	2.0485897064209
FABP6	1.98931562900543
FADS1	1.97097682952881
ACSL1	1.73572699102896
SEMA3C	1.72525085686867
LONP2	1.47963955172447

IDE	1.45531608775552
ALDH9A1	1.44688725471497
CRABP1	1.44518518447876
NR1I2	1.4104517698288
ECI2	1.31663165681787
CTPS1	1.30026899642072
YWHAH	1.18796241283417
PEX6	1.18560476630797
MVP	1.17668211460114
ABCB1	1.12266480922699
DLG4	0.959067106246948
ATXN1	0.95817244052887
PEX5	0.914699076559399
ABCD2	0.888072848320007
ABCC5	0.869463324546814
HRAS	0.839134971993951
CTBP1	0.819535734044322
TSPO	0.639193713665009
ACSL5	0.630192832736629
IDH2	0.59225117554574
EPHX2	0.58408540903497
NUDT19	0.491290356768309
CACNA1B	0.491262867277656
ALB	0.463138925188247
VPS4B	0.431010156869888
TTR	0.399503260850906
SULT2B1	0.231715073335768
CLN8	0.131551551428588
RDH11	0.102856811678627
ACOX1	0.0733462043704366

PEX11B	0.070492334663868
SLC25A4	0.0358498468995094
SLC25A17	0.0358004282490421
HSD17B11	0.0162292336331964
BCL10	-0.00241431292544185
ABCD3	-0.039922684431076
HMGCL	-0.0734709141387598
CDK7	-0.0790873616933823
SCP2	-0.102061940059026
GNPAT	-0.10984994218952
DHRS3	-0.120071083307266
CRABP2	-0.192239640746695
HSD3B7	-0.192381531000137
ISOC1	-0.194867442135236
PRDX5	-0.257052481174469
PEX11A	-0.318717662768062
ABCB4	-0.321846929709755
MLYCD	-0.329452773788221
ERCC3	-0.362680524587631
HSD17B4	-0.408148258924484
ECH1	-0.411122857250775
ELOVL5	-0.498216636857585
SLC35B2	-0.645164489746094
SMARCC1	-0.671024193154455
IDH1	-0.672808706760406
ABCC8	-0.815831466189483
ITGB1BP1	-0.926862321708576
CNBP	-0.961186528205872
ACOT8	-1.05210288126392
SLC27A2	-1.05491077479041

SOD1	-1.09255468845367
PEX2	-1.18128180503845
ABCB9	-1.27028942108154
ACAA1	-1.64269626140594
FIS1	-1.64325511455536
CRAT	-1.89146685600281
